# Burden and Mortality Outcomes of *Clostridioides difficile* Infection Among Patients with Chronic Obstructive Pulmonary Disease: Findings from a Nationwide Database

**DOI:** 10.3390/jcm15114110

**Published:** 2026-05-26

**Authors:** Chloe Lahoud, Daniel Kalta, John Afif, Aysan Sattarzadeh, Faris Qaqish, Tamara Merhej, Rabindra Dhakal, Suzanne El-Sayegh

**Affiliations:** 1 Department of Internal Medicine, Staten Island University Hospital, Northwell Health, 475 Seaview Avenue, Staten Island, NY 10305, USA; 2 Department of Internal Medicine, Mount Sinai Morningside/West, New York, NY 10025, USA; 3 Department of Nephrology, Staten Island University Hospital, Northwell Health, 475 Seaview Avenue, Staten Island, NY 10305, USA

**Keywords:** *Clostridioides difficile*, chronic obstructive pulmonary disease, healthcare utilization

## Abstract

**Background/Objectives**: *Clostridioides difficile* infection (CDI) is the leading cause of colitis and hospital-acquired diarrhea. Patients with Chronic Obstructive Pulmonary Disease (COPD) frequently have infectious exacerbations requiring treatment with antibiotics, which may be predisposing them to CDI. This study examines the prevalence and in-hospital outcomes of CDI in patients with COPD. **Methods**: Data for hospitalized patients with CDI was extracted from the National Inpatient Sample database for the years 2016 through 2020. Baseline risk factors were identified using the International Classification of Diseases codes. Patients were stratified into two groups: with COPD and without COPD. The primary outcome was in-hospital mortality. The secondary outcomes were septic shock, hypovolemic shock, AKI, cardiac arrest, need for intensive care unit (ICU) level of care and length of stay. Statistical analyses were conducted using SPSS. **Results**: 290,172 patients were included in this study. Patients with COPD had more comorbidities overall and higher in-hospital mortality rates compared to patients without COPD (7.7% vs. 5.9%, *p* < 0.001). On multivariate logistic regression analysis, patients with CDI and COPD had higher risk of in-hospital mortality (OR = 1.346, *p* < 0.001), septic shock (OR = 1.289, *p* < 0.001), hypovolemic shock (OR = 1.184, *p* < 0.001), cardiac arrest (OR = 1.362, *p* < 0.001) and required more ICU level of care. **Conclusions**: Patients with COPD experience frequent exacerbations, often requiring hospitalizations and broad-spectrum antibiotics, steroids, proton pump inhibitors and antacids. These factors contribute to the higher prevalence of CDI in this patient population. Patients with CDI and COPD are also more likely to require ICU level of care, shedding the light on the significant burden of CDI, long hospital stays and substantial hospital charges. Recognizing mortality outcomes is essential to guide patient-specific therapies and highlights the need for closer monitoring and targeted management of CDI in patients with COPD.

## 1. Introduction

*Clostridioides difficile* is an obligate anaerobic, spore-forming, Gram-positive bacillus that produces toxins responsible for gastrointestinal disease, most commonly presenting as diarrhea. It is recognized as the most common cause of hospital-acquired infectious diarrhea and represents a significant burden on healthcare systems. Although historically associated with healthcare settings and antibiotic exposure, *Clostridioides difficile* is increasingly recognized in the community [1]. This rise has been attributed in part to widespread antibiotic use and the emergence of hypervirulent and antibiotic-resistant strains such as ribotype 027, also known as the North American pulsed-field gel electrophoresis type 1 (NAP1) strain [2]. Clinical manifestations of *Clostridioides difficile* colonization range from asymptomatic carriage to severe outcomes such as pseudomembranous colitis, toxic megacolon, and septic shock. Asymptomatic colonization can disrupt the gut microbiome and predispose individuals to active infection, particularly following antibiotic exposure.

CDI is associated with substantial morbidity, including prolonged hospital stays, increased healthcare costs, and mortality [3,4,5]. Broad-spectrum antibiotic exposure remains the most significant risk factor for CDI [6]. Additional risk factors include advanced age, immunosuppression, proton pump inhibitor use, prior CDI, recent hospitalization, prolonged hospital stay, and underlying comorbidities [7,8,9]. Among these comorbidities, COPD has been increasingly recognized as a condition associated with a higher risk of CDI [10,11,12].

Patients with COPD frequently require hospitalization and antibiotic therapy, particularly during acute exacerbations, both of which are established risk factors for CDI. Antibiotics commonly used in this population—including penicillins, cephalosporins, fluoroquinolones, and clindamycin—have well-documented associations with CDI development [13,14]. Proposed mechanisms include frequent antibiotic exposure, recurrent hospitalizations, and a higher burden of comorbidities in this population. These factors may place patients with COPD at particularly elevated risk for CDI and its complications.

Despite this potential vulnerability, most of the existing evidence is derived from smaller or single-center studies. There remains limited large-scale data examining the burden, trends, and outcomes of CDI among hospitalized patients with COPD [15]. Therefore, using a nationwide database, this study aims to evaluate the prevalence and in-hospital outcomes of CDI among patients with COPD, compared to patients without COPD.

## 2. Materials and Methods

### 2.1. Overview of the Nationwide Inpatient Sample

The Nationwide Inpatient Sample (NIS) is a database within the Healthcare Cost and Utilization Project (HCUP) sponsored by the Agency for Healthcare Research and Quality (AHRQ). It is a large administrative database representing inpatient hospitalizations across the United States. In this study, the use of the NIS enables a nationwide, population-level analysis, thereby enhancing the generalizability and novelty of the findings, especially when compared to single-center or regional studies. The NIS is the largest publicly accessible inpatient database in the country and is commonly used to generate national and regional estimates related to healthcare utilization, access to care, costs, quality, and clinical outcomes. The NIS contains information from roughly seven million hospital stays annually. Because of its large sample size and standardized data collection, the database provides a robust source for national-level analyses. It includes detailed hospitalization data such as patient demographics, diagnoses, procedures performed, and hospital characteristics. Each hospitalization is recorded as a separate entry in the dataset, meaning that the same individual may appear multiple times if admitted to the hospital on more than one occasion.

### 2.2. Data Source and Variables of Interest

Diagnoses and comorbid conditions pertinent to the analysis were identified using codes from the International Classification of Diseases, Tenth Revision (ICD-10). Hospitalizations involving patients younger than 18 years of age were excluded from the study population. Because the NIS database contains fully de-identified patient information, the study qualified for exemption from review by the Institutional Review Board (IRB) at Northwell Health.

### 2.3. Study Design

A cross-sectional design was employed using data from the NIS database, which provides access to a large, nationally representative sample of inpatient hospitalizations in the United States. The extensive size of this dataset makes it particularly useful for investigating less common conditions that require large populations to achieve sufficient statistical power.

Data for hospitalized patients with CDI was extracted from the NIS for the years 2016 through 2020. The study period was selected based on the availability of data through our institution. Cases were included whether CDI was a primary diagnosis or in any diagnosis position. Discharge weights were not used in this study. Diagnoses and baseline comorbidities were identified using ICD-10 codes. The study population included adult patients aged 18 years or older with a recorded diagnosis of CDI. ICD-10 codes were used to identify patients with COPD (both acute and chronic COPD). These hospitalizations were then categorized into two cohorts: patients with COPD and patients without COPD.

Demographic variables including age, sex, and race were collected, along with socioeconomic and hospital-related factors such as hospital location, primary insurance payer, and median household income by ZIP code. These variables are available within the NIS as standardized administrative data elements recorded during the hospital admission process. The analysis included multiple comorbid conditions such as hypertension (HTN), diabetes mellitus (DM), dyslipidemia (DLD), overweight (defined as a Body Mass Index (BMI) between 25 and 29.9), obesity (defined as a BMI > 30), alcohol use, smoking use, chronic kidney disease (CKD) stages 3 to 5, end stage renal disease (ESRD), and coronary artery disease (CAD). Moreover, ICU-level care was defined by the presence of procedures or interventions indicative of critical illness, including intubation and mechanical ventilation, central venous catheter placement, arterial line placement, or vasopressor use, as identified using ICD-10 codes. The list of ICD-10 codes used is provided in Appendix A Table A1.

In-hospital mortality was designated as the primary outcome. Secondary endpoints were toxic megacolon, AKI, hypovolemic shock, septic shock, need for total parenteral nutrition (TPN), cardiac arrest, need for blood transfusion, need for ICU level of care and length of stay.

### 2.4. Statistical Analysis

The collected data was coded and statistically analyzed using IBM Statistical Software (Version 30.0; IBM Corp., Chicago, IL, USA).

Age and LOS are reported as mean values with their corresponding standard errors, and the Wilcoxon test was employed to compare the two groups. Categorical variables are expressed as percentages accompanied by standard errors and were analyzed using the second-order Pearson’s chi-square test. The analysis utilized the complex sampling capabilities of Statistical Package for Social Sciences (SPSS) Statistics V.30.

Categorical variables were described as frequencies and percentages. Multivariate binary logistic regression was performed to evaluate the independent association between COPD and the outcomes of interest, while adjusting for relevant demographic and clinical covariates. Results are reports as odds ratios (OR) with 95% confidence intervals. Statistical significance was defined as a *p*-value < 0.05.

## 3. Results

This study included 290,172 patients hospitalized with CDI, among which 57,351 (19.7%) had COPD. Several demographic and clinical characteristics were significantly different between patients without COPD compared to patients with COPD, as shown in Table 1.

Patients with COPD were notably older than those without COPD (mean age 70.8, compared to 65 years). There was a higher prevalence of female patients in both groups (57.1 and 58.2% in patients without and with COPD respectively). The racial distribution data indicated that white patients comprised the majority in both groups (71.8% in patients without COPD and 81.6% in patients with COPD). Medicare was the most common primary expected payer (62.6% in patients without COPD and 77.5% in patients with COPD). In both groups, the majority of patients were female and white, and Medicare was the most common primary payer, reflecting the older age of the hospitalized population with CDI. The population setting and median household income are listed in Table 1.

There was a significantly higher burden of comorbid conditions including DM, HTN, DLD, CKD stages 3–5 and CAD in the group of patients with COPD. Less patients with COPD were overweight (2.5% compared to 3% in patients without COPD); however, more patients were obese (17.3% compared to 14% in patients without COPD). Patients with COPD were more frequently smokers than patients without COPD (34.1% compared to 20.1% in patients without COPD), consistent with the known epidemiology of the disease. Additional demographic and socioeconomic characteristics are presented in Table 1.

Among patients admitted with CDI, patients with COPD had a statistically significant higher in-hospital mortality rate compared to patients without COPD (7.7% vs. 5.9%, *p*-value < 0.001). Patients with COPD had a slightly longer length of stay; however, the difference was not found to be statistically significant (10.06 vs. 9.97, *p*-value = 0.162). Moreover, patients with COPD had slightly lower total hospital charges when compared to patients without COPD ($102,220 vs. $105,689), although the absolute difference was modest despite reaching statistical significance (*p* < 0.001) (Table 2). To note, hospital charges were reported as provided in the database and were not adjusted for inflation across the study years.

The multivariate regression analysis is presented in Table 3 and is adjusted for patient demographic and clinical characteristics. This model evaluated whether the presence of COPD was independently associated with an increased risk of each of the listed secondary outcomes, among patients hospitalized with CDI.

COPD was independently associated with a higher risk of in-hospital mortality (OR = 1.35, *p*-value < 0.001), hypovolemic shock (OR = 1.18, *p*-value < 0.001), septic shock (OR = 1.28, *p*-value < 0.001), cardiac arrest (OR = 1.36, *p*-value < 0.001), need for blood transfusion (OR = 1.1, *p*-value < 0.001) and need for ICU level of care (intubation (OR = 1.79, *p*-value < 0.001), central line (OR = 1.30, *p*-value < 0.001), arterial line (OR = 1.36, *p*-value < 0.001), and vasopressors (OR = 1.19, *p*-value < 0.001) (Table 3). There was no statistically significant association between COPD and the development of toxic megacolon or AKI in the adjusted regression analysis (Table 3).

## 4. Discussion

In this study of a large nationally representative sample, the prevalence rates of hospitalizations for CDI and the in-hospital mortality rates in COPD patients were higher than in patients without COPD. Patients with COPD experience frequent exacerbations, often require hospitalizations, and are commonly treated with broad-spectrum antibiotics, systemic corticosteroids, and gastric acid-suppressive therapies, including proton pump inhibitors and antacids. All these factors could explain the higher prevalence of CDI in this patient population, when compared to patients without COPD. Additionally, COPD is commonly associated with a high burden of chronic comorbidities, which may further increase susceptibility to infectious and adverse outcomes. Results show that patients with COPD had more comorbidities overall, including DM, HTN, obesity, CKD, and CAD, which is consistent with published literature.

Persistent inflammation, endothelial dysfunction and hypoxia are all COPD-specific mechanisms which promote atherosclerosis and vascular disease. As a result, patients with COPD are at a two to five times more risk of developing ischemic heart disease, cardiac arrhythmia, heart failure, pulmonary vascular diseases, and arterial disease [16]. Current medical literature discusses the correlation between beta blocker usage in COPD and infection risk presents conflicting information. This is primarily dependent on beta blocker selectivity and the clinical context of its usage. Two major randomized control trials indicate that in patients without cardiovascular risk factors, utilization of beta blockers in COPD did not reduce exacerbations and contributed to increased risk of hospitalization [17]. However, there is no medical literature which cites a direct correlation between beta blocker usage and increased risk of CDI. A recently published pharmacologic study observed 16,196 cases of CDI and examined 335 drugs and showed that beta blockers were not associated with an increased CDI risk [18]. Current medical literature does not clinically reference a relationship between blocker utilization in COPD and increased CDI rates; antibiotic exposure and PPI usage remain the primary modifiable risk factors for CDI in COPD patients.

The substantial inflammatory burden, presence of chronic comorbidities, frequent healthcare exposure and typical management of COPD exacerbations offer important insight into the observed findings. Patients with COPD exacerbations were found to have a 2.24-fold higher increase in CDI when compared to age-, sex-, and year-matched control groups [19]. This increased susceptibility is potentially correlated with prolonged hospitalizations, systemic inflammation, and prolonged antibiotic exposure in COPD patients. Patients with COPD and concomitant cardiovascular disease develop a synergistically increased risk of CDI. This correlation is attributed to similar pathophysiologic mechanisms between both processes. Mechanisms such as endothelial cell dysfunction, oxidative stress, and integration of inflammatory markers including IL-6, Fibrinogen and TNF-alpha all contributed to increased CDI risk [20,21]. Furthermore, patients with COPD along with cardiovascular disease have been found to have generally longer hospitalization courses, increased antibiotic administration, and increased rates of rehospitalization, all of which predispose to increased infection risk [22]. The mortality risk of patients with CDI and concomitant cardiovascular disease warrants intensive antibiotic stewardship and clinical surveillance, which can effectively contribute towards a reduction in infection rates and inpatient complications.

Renal disease may also play an important role in this association. COPD has been shown to independently increase the risk of CKD. A meta-analysis showed a 50–60% increased risk of CKD in COPD patients, and renal function declines more rapidly in this population [23]. Worsening chronic kidney disease complicates antibiotic prescribing by restricting therapeutic options and necessitating dose adjustments, thereby increasing the complexity of achieving appropriate dosing and potentially prolonging systemic antibiotic exposure. CKD alone is an independent risk factor for both initial and recurrent CDI, nearly doubling the risk compared to patients without CKD. The pathophysiologic mechanism of compounded CDI risk in this patient population is similar to that of patients with COPD and concomitant cardiovascular disease. Such factors include recurrent hospitalizations, systemic inflammation, and prolonged PPI usage. The compounded effect of COPD induced immunologic dysregulation and CKD induced uremic dysfunction both contribute towards an imbalance of the gut microbiome (gut dysbiosis) which exacerbates CDI risk [24,25]. Given the prevalence of CKD in patients with COPD, incorporation of standardized antibiotic regimens specifically tailored to renal function is an intricate balance, as the risk of CDI is a prominent complication seen in these patient populations.

The GOLD criteria provide limited support for routine antibiotic use for COPD exacerbations, noting low certainty regarding the overall benefit of antibiotics in some clinical scenarios. Therefore, clinicians should exercise caution when prescribing antibiotics for COPD exacerbations, particularly in the absence of clear bacterial infection, as unnecessary exposure may increase the risk of CDI [26].

Patients with CDI and COPD are also more likely to require ICU level of care. This aligns with the published literature shedding the light on the significant clinical burden of CDI, which includes high rates of re-hospitalizations, prolonged hospital stays as well as substantial hospital charges. However, in our findings, the difference in LOS was not statistically significant between the two groups. This finding may reflect evolving CDI management strategies over time, which may have improved treatment efficacy and potentially mitigated differences in hospital utilization. As per ACG guidelines, oral vancomycin and fidaxomicin can both be used as first-line therapy for treatment of non-severe and severe CDI. For fulminant CDI, parenteral metronidazole can be added to therapy. Fecal microbiota transplantation, via colonoscopy or capsule, is recommended for severe/fulminant CDI that is refractory to antibiotic therapy and for recurrence of CDI (within 8 weeks of initial treatment). For CDI refractory to fecal microbiota transplant (FMT), suppressive oral vancomycin is recommended long-term for the management of CDI [22]. These are significantly different from the 2013 recommended strategies, prior to fidaxomicin and FMT [27]. These changes may have contributed to the improved outcomes and allowed LOS to remain unchanged between the two groups.

## 5. Limitations and Future Directions

This manuscript highlights an important gap in existing knowledge, emphasizing the need for a tailored approach to address these challenges and improve outcomes in this patient population. Major strengths of this study include the large cohort size, utilization of nationally representative data, and comprehensive analysis of risk factors.

Nonetheless, the study has several limitations. The cross-sectional nature of the study limits the ability to determine temporal relationships. Additionally, the database lacks granular information regarding disease activity (acute or chronic COPD, oxygen use), medication history (such as steroid use), and microbiological or laboratory information. Therefore, subgroup analyses based on medication use were not feasible due to limitations of the database. The use of ICD-10 codes to identify variables of interest relies on accurate provider documentation and is subject to potential coding errors. Additionally, because the NIS records hospitalizations as separate entries, individual patients may be represented more than once; however, this likely has a minimal effect given the large sample size. Finally, the database includes only hospitalized individuals with COPD, which may limit the generalizability of the findings to the broader COPD population. Additionally, despite adjustment for multiple demographic and clinical variables, residual confounding may still be present due to the observational nature of the study and the limitations of administrative databases.

Prospective longitudinal studies should be prioritized in future research efforts to identify modifiable risk factors that may improve patient outcomes and management strategies.

## 6. Conclusions

This nationwide, large-scale database analysis highlights a significant CDI burden in hospitalized patients with COPD, characterized by higher in-hospital mortality and a greater need for intensive-care-level interventions compared with patients without COPD. These findings emphasize the vulnerability of COPD patient populations to severe CDI-associated outcomes, primarily reflective of the combined effects of comorbidity burden, antibiotic exposure, chronic systemic inflammation, acid-suppressive therapies, and corticosteroid administration in COPD exacerbations. Such associations highlight the necessity of early recognition/treatment of CDI and implementation of antibiotic stewardship in hospitalized patients with COPD. Consideration of increased infectious risk and optimization of comorbidities may help decrease adverse outcomes associated with CDI in this patient population. Future prospective studies should be utilized to identify modifiable risk factors and in turn develop targeted prevention strategies aimed at improving outcomes in this high-risk population.

## Figures and Tables

**Table 1:** Demographics and clinical characteristics of included patients admitted with CDI between 2016 and 2020 (*n* = 290,172). A *p*-value below 0.05 is considered statistically significant.

Demographics	No COPD	COPD	*p*-Value
Total *n* (%)	232,821 (80.3)	57,351 (19.7)	
Age (mean, SD)	65, 17.56	70.84, 11.58	<0.001
Sex female (%)	132,823 (57.1)	33,367 (58.2)	<0.001
**Race**			<0.001
White	162,319 (71.8)	45,590 (81.6)	
Black	31,047 (13.7)	5922 (10.6)	
Hispanic	20,726 (9.2)	2530 (4.5)	
Asian	4713 (2.1)	575 (1)	
Native American	1749 (0.8)	277 (0.5)	
Other	5526 (2.4)	988 (1.8)	
**Primary expected payer**			<0.001
Medicare	145,523 (62.6)	44,392 (77.5)	
Medicaid	29,715 (12.8)	5559 (9.7)	
Private	46,508 (20)	5620 (9.8)	
Self-pay	5587 (2.4)	621 (1.1)	
None	498 (0.2)	57 (0.1)	
Other	4471 (2)	1044 (1.8)	
**Population Setting**			<0.001
>1 million in central city	66,578 (28.7)	14,130 (24.7)	
>1 million fringe of city	57,459 (24.8)	13,756 (24.1)	
250 K–1 million population	48,671 (21)	12,062 (21.1)	
50 K–250 K population	22,622 (9.8)	6364 (11.1)	
<50 K Metropolitan counties	21,197 (9.1)	6298 (11)	
<50 K Not metropolitan counties	15,310 (6.6)	4529 (7.9)	
**Median Household Income**			<0.001
0–25th percentile	63,621 (27.8)	18,069 (32)	
26–50th percentile	60,572 (26.5)	16,050 (28.5)	
51–75th percentile	56,329 (24.6)	13,011 (23.1)	
76–100th percentile	48,474 (21.2)	9280 (16.5)	
DM	77,730 (33.4)	21,200 (37)	<0.001
HTN	156,437 (67.2)	44,415 (77.4)	<0.001
Dyslipidemia	60,924 (26.2)	19,177 (33.4)	<0.001
Overweight BMI 25 to 29.9	6869 (3)	1430 (2.5)	<0.001
Obesity BMI 30+	32,536 (14)	9927 (17.3)	<0.001
Alcohol	15,390 (6.6)	4456 (7.8)	<0.001
Smoking	46,846 (20.1)	19,533 (34.1)	<0.001
CKD 3–5	33,951 (14.6)	10,653 (18.6)	<0.001
ESRD	23,005 (9.9)	5338 (9.3)	<0.001
CAD	47,815 (20.5)	19,744 (34.4)	<0.001

**Table 2:** In-hospital mortality, length of stay and total hospital charges in patients admitted for CDI.

	No COPD	COPD	*p*-Value
**Mortality: *n* (%)**	13,662 (5.9)	4399 (7.7)	<0.001
**Length of Stay: Mean, SD**	9.97, 13.29	10.06, 11.73	0.162
**Total hospital charges: Mean, SD**	105,689.30; 216,342.585	102,220.97; 173,429.706	<0.001

**Table 3:** Multivariate regression for CDI risk in patients with COPD. Adjusted for age, sex, race, and comorbidities (DM, HTN, DLD, overweight, obesity, CKD stages 3 to 5, ESRD, smoking and alcohol).

	OR	Lower CI 95%	Higher CI 95%	*p*-Value
In-hospital mortality	1.346	1.297	1.397	<0.001
Toxic Megacolon	1.365	0.754	2.471	0.304
AKI	1.016	0.995	1.037	0.138
Hypovolemic shock	1.184	1.089	1.287	<0.001
Septic Shock	1.289	1.251	1.329	<0.001
TPN	0.981	0.868	1.109	0.755
Cardiac Arrest	1.362	1.267	1.465	<0.001
Blood transfusion	1.1	1.064	1.137	<0.001
Intubation	1.798	1.745	1.853	<0.001
Central line	1.305	1.241	1.373	<0.001
Arterial line	1.368	1.25	1.498	<0.001
Vasopressors	1.194	1.121	1.271	<0.001

## Data Availability

The data supporting the findings of this study are obtained from HCUP. Access to these data is subject to licensing restrictions and was granted for the purposes of this analysis. Data may be made available by the authors upon reasonable request and with permission from HCUP.

## Abbreviations

The following abbreviations are used in this manuscript:

AHRQ	Agency for Healthcare Research and Quality
AKI	Acute Kidney Injury
BMI	Body Mass Index
CAD	Coronary Artery Disease
CDI	*Clostridioides difficile* Infection
CI	Confidence Interval
CKD	Chronic Kidney Disease
COPD	Chronic Obstructive Pulmonary Disease
DM	Diabetes Mellitus
DLD	Dyslipidemia
ESRD	End-Stage Renal Disease
FMT	Fecal Microbiota Transplant
GOLD	Global initiative for chronic Obstructive Lung Disease
HCUP	Healthcare Cost and Utilization Project
HTN	Hypertension
ICD-10	International Classification of Diseases, 10th Edition
ICU	Intensive Care Unit
IRB	Institutional Review Board
LOS	Length Of Stay
NAP1	North American pulsed-field gel electrophoresis type 1 strain
NIH	National Institute of Health
NIS	Nationwide Inpatient Sample
OR	Odds Ratio
SD	Standard Deviation
SPSS	Statistical Package for Social Sciences
TPN	Total Parenteral Nutrition

## Appendix A

**Table A1:** ICD-10 codes used.

CDI	A047
COPD	J41, J42, J43, J44
Diabetes mellitus	E08, E09, E10, E11, E13
Hypertension	I10
Dyslipidemia	E780, E781, E782, E783, E784, E785
Overweight BMI 25 to 29.9	Z6825, Z6826, Z6827, Z6828, Z6829, E663
Obesity BMI 30+	E6601, E6609, E661, E662, E668, E669, O99210, O99211, O99212, O99213, O99214, O99215, R939, Z6830, Z6831, Z6832, Z6833, Z6834, Z6835, Z6836, Z6837, Z6838, Z6839, Z6841, Z6842, Z6843, Z6844, Z6845, Z6854
Alcohol	F10
Smoking	F17200, Z87891
CKD 3–5	N183, N184, N185
ESRD	N186
Coronary artery disease	I2510, I252, I258, I259
Toxic megacolon	K5931
AKI	N17
Hypovolemic shock	R571
Septic shock	R6521
TPN	3E0336Z
Cardiac arrest	I462, I468, I469
Intubation	0BH17EZ, 0BH18EZ, 5A1935Z, 5A1945Z, 5A1955Z
Arterial line	4A133B1
Central line	06HN33Z, 06HM33Z, 05H633Z, 05H533Z, 05HN33Z, 05HM33Z, 05HM3DZ
Vasopressor	3E033XZ, 3E043XZ
Blood transfusion	30233N1
